# Ultrahigh strength and ductility in newly developed materials with coherent nanolamellar architectures

**DOI:** 10.1038/s41467-020-20109-z

**Published:** 2020-12-07

**Authors:** Lei Fan, Tao Yang, Yilu Zhao, Junhua Luan, Gang Zhou, Hao Wang, Zengbao Jiao, Chain-Tsuan Liu

**Affiliations:** 1 Department of Mechanical Engineering, The Hong Kong Polytechnic University, Hong Kong, China; 2 Department of Materials Science and Engineering, City University of Hong Kong, Hong Kong, China; 3 Shi-changxu Innovation Center for Advanced Materials, Institute of Metal Research, Chinese Academy of Sciences, Shenyang, 110016 China

**Keywords:** Mechanical properties, Metals and alloys

## Abstract

Nano-lamellar materials with ultrahigh strengths and unusual physical properties are of technological importance for structural applications. However, these materials generally suffer from low tensile ductility, which severely limits their practical utility. Here we show that markedly enhanced tensile ductility can be achieved in coherent nano-lamellar alloys, which exhibit an unprecedented combination of over 2 GPa yield strength and 16% uniform tensile ductility. The ultrahigh strength originates mainly from the lamellar boundary strengthening, whereas the large ductility correlates to a progressive work-hardening mechanism regulated by the unique nano-lamellar architecture. The coherent lamellar boundaries facilitate the dislocation transmission, which eliminates the stress concentrations at the boundaries. Meanwhile, deformation-induced hierarchical stacking-fault networks and associated high-density Lomer-Cottrell locks enhance the work hardening response, leading to unusually large tensile ductilities. The coherent nano-lamellar strategy can potentially be applied to many other alloys and open new avenues for designing ultrastrong yet ductile materials for technological applications.

## Abstract (web summary)

Nano-lamellar materials with ultrahigh strengths are highly desirable for technological applications. Here the authors report a nanolamellar architecturing approach by utilizing coherent L12 structures to achieve ultrahigh strength and ductility in Ni-Fe-Co-Cr-Al-Ti multicomponent alloys.

## Introduction

Advanced structural materials with ultrahigh strengths and excellent ductilities are highly desirable for a wide variety of technological applications, including aerospace, transportation, and energy sectors. Nanolamellar alloys containing high-density interfaces are of particular interest owing to their unusual interface-driven properties, such as extremely high strengths^1–8^. Unfortunately, these materials typically suffer from a severe limitation—lack of tensile ductility, typically <5% elongation, and the region of the uniform deformation is even more limited^9–12^. This makes them difficult to be fabricated and prone to catastrophic failure in load-bearing applications. The strength-ductility trade-off has been a long-standing dilemma, which has attracted great attention in materials science^13–17^. Recently, several engineered heterogeneous nanostructures, such as gradient, bimodal, harmonic, and hierarchical structures, have been demonstrated to have the potential to enhance tensile ductility of nanostructured materials, leading to improved strength-ductility synergy^18–23^. However, these approaches usually produce materials with limited strengths (on the order of 1 GPa) because the creation of microstructural heterogeneity requires the incorporation of some coarse-scale microstructures that sacrifice the strength. By contrast, for nanolamellar alloys with extremely high strengths on the order of 2 GPa, brittleness remains their Achilles’ heel, which can be ascribed to the absence of work-hardening and serious strain localization at incoherent lamellar boundaries. Furthermore, most nanolamellar alloys are fabricated in the form of thin films/sheets by such methods as electrodeposition and severe plastic deformation^1^, which are not readily applicable to the large volume components necessary for practical applications. Consequently, developing ultrahigh strength, ductile, and scalable nanolamellar alloys are highly desirable but extremely challenging.

Here we overcome these critical challenges and present the development of new nanolamellar alloys featuring in situ-formed coherent nanolamellar (CNL) architectures, which exhibit an unprecedented combination of ultrahigh strength and tensile ductility and can be produced through conventional casting and thermomechanical treatments. Our design philosophy is to create nanometer-thick lamellae for an extremely high strengthening purpose while maintaining coherent lamellar boundaries that can suppress stress concentration and accommodate plastic strain, thereby promoting work hardening together with tensile ductility for nanostructured materials. In particular, we chose Ni-Fe-Co-Cr-Al-Ti multicomponent alloys as our model system, because they provide a large dual-phase region consisting of disordered face-centered cubic (FCC) and ordered FCC (L1_2_) phases (Supplementary Fig. 1) with a small lattice mismatch, satisfying the requirement for the formation of coherent boundaries. The CNL architectures were controlled through a solid-state phase separation involving the process of “supersaturated FCC solid solution → L1_2_ + FCC lamellae” within ultrafine grains at a relatively low temperature of 600 °C. At this temperature, the bulk diffusion is significantly inhibited, which kinetically suppresses the continuous precipitation of spheroidal nanoparticles. In contrast, the ultrafine grain structure promotes the discontinuous precipitation of nano-lamellae, because the high-density grain boundaries not only provide numerous nucleation sites but also promote their growth through grain boundary diffusion, which leads to the formation of unique FCC/L1_2_ CNL architecture across the whole grains.

## Results

### Tensile properties

**Fig. 1:**
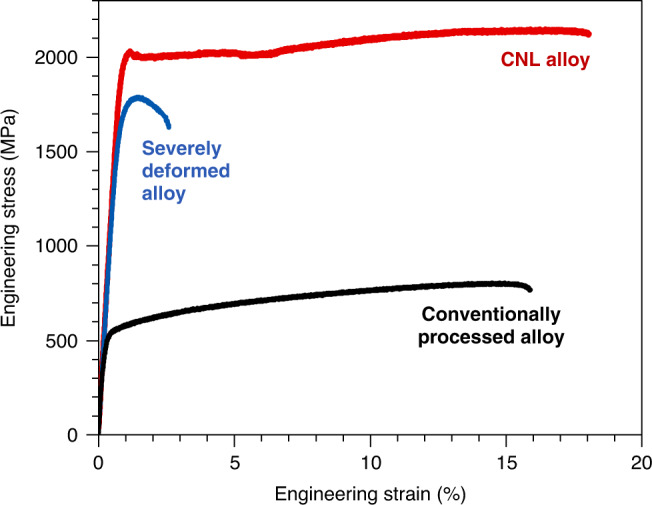
Tensile stress–strain curve of the coherent nanolamellar (CNL) alloy. The curves of the conventionally processed alloy (black) and severely deformed alloy (blue), which have the same composition as the CNL alloy but without the CNL architectures, are presented for comparison. The CNL alloy (red) reveals an extraordinary combination of ultrahigh strength and large ductility.

We performed tensile tests at room temperature to quantitatively measure the mechanical properties of a representative CNL alloy, Ni_32.8_Fe_21.9_Co_21.9_Cr_10.9_Al_7.5_Ti_5.0_ (at.%), which can also be written as (Ni_1.5_FeCoCr_0.5_)_87.5_Al_7.5_Ti_5.0_. To emphasize the substantial improvement in mechanical property upon the formation of the CNL architecture, the curves of two other samples having the same composition as the CNL alloy but without any CNL architectures are presented for comparison. The two reference materials are, respectively, a conventionally processed alloy and a severely deformed alloy (see Methods). From these curves, we see that our CNL alloy exhibits an extraordinary combination of ultrahigh strength and large uniform ductility (Fig. 1). The yield strength reaches as high as 2026 ± 20 MPa, and the ultimate tensile strength to 2118 ± 34 MPa; these values are more than quadruple of those for the conventionally processed alloy without such CNL architectures. More intriguingly, the ultrahigh strength CNL alloy also shows a large tensile ductility, with a uniform elongation of 16%, which is an order of magnitude larger than that of the severe deformed alloy with high dislocation densities (~1% uniform elongation). In addition, from the true stress–strain curve (Supplementary Fig. 2a), an obvious work hardening appears in the major plastic deformation stage, indicative of considerable dislocation accumulation during the plastic straining before failure. The work-hardening rate curve (Supplementary Fig. 2b) reveals a multi-stage work-hardening response. After a precipitous initial drop associated with the elastic to plastic transition, the work-hardening rate increases rapidly to a peak at the 2% strain and then drops gradually till a strain of 5%. Beyond that strain, the work-hardening rate increases again till the 6% strain, followed by a monotonic decrease. The fracture surface has two regions, i.e., the peripheral shear lip and central flat fracture regions (Supplementary Fig. 3). Both regions reveal a microvoid coalescence fracture mode with a plenty of fine dimples, indicating a characteristic mode of a ductile fracture at room temperature. The large area of shear lips indicates that the plane stress state prevails during the fracture, which is related to the small thickness of the tensile specimens. The specimen thickness can influence the post-necking elongation, but it has a negligible impact on the uniform elongation. Thus, our CNL alloy achieves an unprecedented synergy of both ultrahigh strength and large uniform ductility, which are inaccessible to conventional lamellar materials.

### Microstructural characterization

**Fig. 2:**
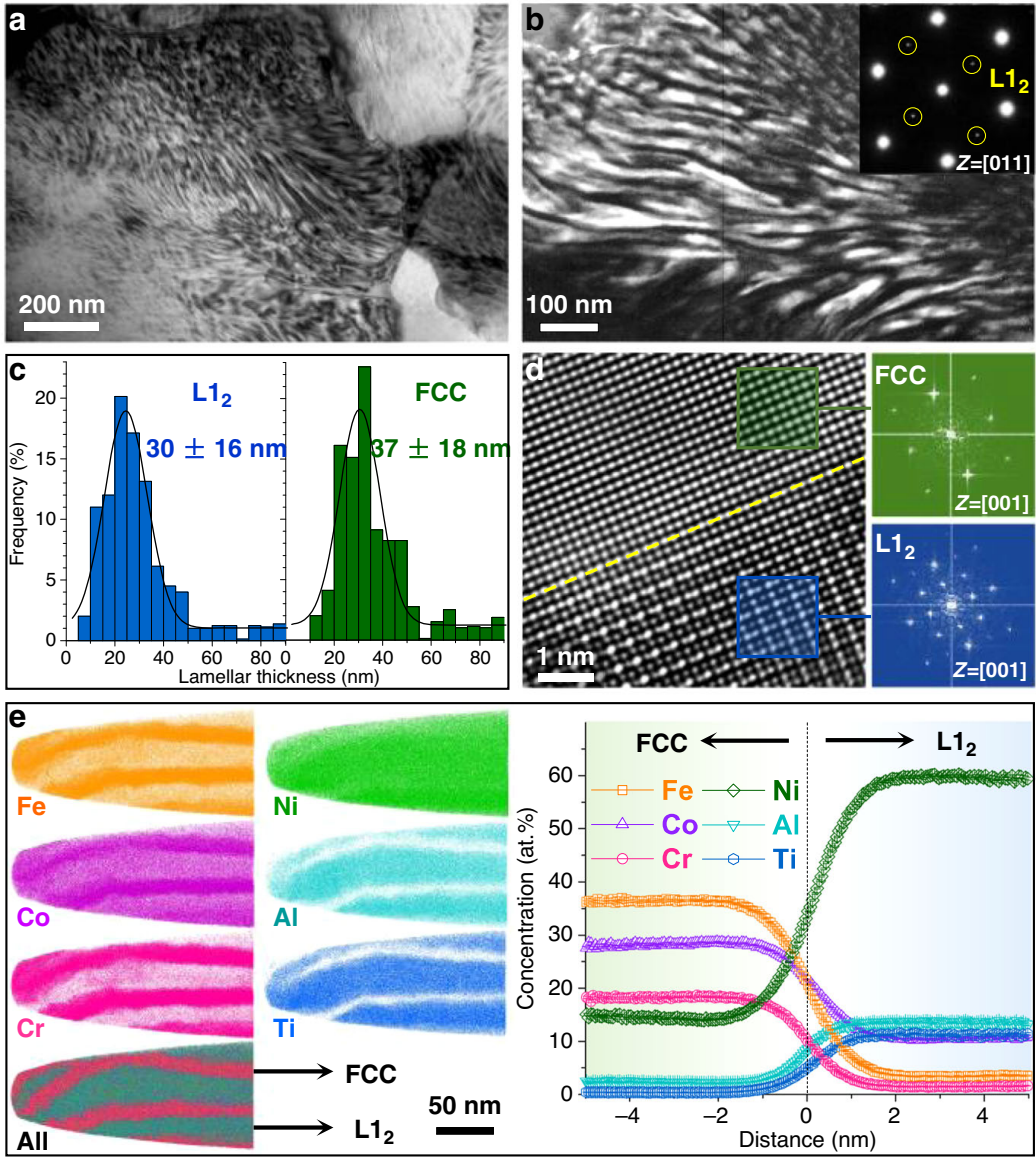
Unique nanolamellar architecture of the CNL alloy. **a**, **b** Bright-field and dark-field TEM microstructure of the FCC and L1_2_ nano-lamellae within ultrafine grains. **c** Statistical distributions for thicknesses of the FCC and L1_2_ nano-lamellae. **d** High-resolution TEM image illustrating the interfacial coherency of the FCC/L1_2_ lamellar boundary. **e** APT characterization of the elemental partitioning and composition of the FCC and L1_2_ nano-lamellae.

To understand the observed superb mechanical properties of the CNL alloy, we studied the underlying microstructures in detail down to the atomic scale. Electron backscatter diffraction (EBSD) reveals that the grain structure exhibits a uniform distribution of ultrafine equiaxed grains with an average size of 390 nm and random orientations along the rolling, transverse, and normal directions (Supplementary Fig. 4), indicating a full recrystallized and uniform microstructure. Each grain contains a high density of alternating lamellae of the order-L1_2_ and disorder-FCC phases with thicknesses in the nanometer range, as observed in bright-field (Fig. 2a) and dark-field (Fig. 2b) transmission electron microscopy (TEM). All the grains have the FCC/L1_2_ nanolamellar structure without any spheroidal L1_2_ nanoparticles. The average thickness for the FCC and L1_2_ lamellae was measured to be ~37 ± 18 and 30 ± 16 nm, respectively (Fig. 2c). From high-resolution TEM (Fig. 2d), we observed a coherent FCC/L1_2_ interface with continuous crystal lattices, where the crystal lattice of the ordered L1_2_ phase is very close to that of the disordered FCC phase. This agrees with the X-ray diffraction (XRD) results that the lattice mismatch between the two phases is only ~0.13% (Supplementary Fig. 5), the small value of which effectively stabilizes the CNL structure without any heterogeneous coarsening. We further analyzed the phase composition and elemental distribution of the FCC/L1_2_ CNL structure by three-dimensional atom probe tomography (3D-APT) (Fig. 2e) and energy-dispersive X-ray spectroscopy mapping in the scanning-TEM mode (STEM-EDS) (Supplementary Fig. 6). We observed that Ni (green), Ti (blue), and Al (cyan) partition to the L1_2_ lamellae, whereas Co (purple), Cr (pink), and Fe (orange) partition to the FCC lamellae. The 12% (Al + Ti) concentration isosurfaces were used to visualize the boundary between the FCC and L1_2_ lamellae, and the corresponding proximity histogram showing the concentration profiles across the boundary is also displayed in Fig. 2e. Data points within the flat region of the profiles at the left and right sides of the interface delineate the composition of the FCC and L1_2_ nano-lamellae, respectively. The composition of the FCC lamellae is 36.4Fe-28.1Co-14.6Ni-18.2Cr-2.3Al-0.4Ti (at.%), whereas that the L1_2_ lamellae can be regarded as 59.7Ni-10.6Co-3.5Fe-1.5Cr-13.5Al-11.2Ti (at.%), yielding a (Ni + Co + Fe + Cr):(Al + Ti) ratio of ~3:1 (A_3_B-type). By using the lever rule analysis, the volume fraction of the FCC and L1_2_ phases are determined to be ~56% and 44%, respectively (Supplementary Fig. 7). From the aforementioned observations, we can see that our newly developed CNL alloy exhibits a unique lamellar architecture consisting of alternating order-L1_2_ and disorder-FCC lamellae with nanometer thicknesses and coherent boundaries, which is significantly distinctive to conventional lamellar alloys with coarse lamellar thickness and/or incoherent boundaries.

Microstructures of the conventionally processed and severely deformed alloys were examined for comparison (Supplementary Fig. 8). The conventionally processed alloy exhibits a mixed microstructure containing spheroidal nanoparticles in grain interiors and nano-lamellae near grain boundaries, which is similar to that reported in the literature. The severely deformed alloy exhibits a typical cellular microstructure containing elongated sub-grains.

## Discussion

We attribute the ultrahigh yield strength of the CNL alloy mainly to the lamellar boundary strengthening associated with nanometer-thick order/disorder lamellae. In this material, the ordered L1_2_ lamellae are much stronger than the FCC solid solution lamellae, because the ordered L1_2_ superlattice structure has high anti-phase boundary energy (~200 mJ m^-2^)^24^, requiring high stresses for dislocations to cut through. Thus, the FCC/L1_2_ lamellar boundaries are strong barriers to the dislocation motion. It was documented that a Hall–Petch relationship also describes the yield strength of dual-phase nanolamellar structures^25^. In the FCC/L1_2_ CNL architecture, initial plastic deformation occurs in the soft FCC lamellae; thus, the parameter controlling the yield strength is the thickness of FCC lamellae. Based on the Hall–Petch modeling, we estimated that the contribution of nanolamellar boundaries to the yield strength is ~1.0 GPa (see Methods), which provides the dominant contribution to the macroscopic yield strength. In addition, the ultrafine grains with an average size of 390 nm also contributes to a large component of the yield strength (0.8 GPa), as estimated from the Hall–Petch relation for grain boundary strengthening. Overall, the collective strengthening contributions from the FCC/L1_2_ nano-lamellae, ultrafine grain sizes, together with other strengthening from solid solution and dislocations, elevate the yield strength of the CNL alloy beyond 2 GPa.

**Fig. 3:**
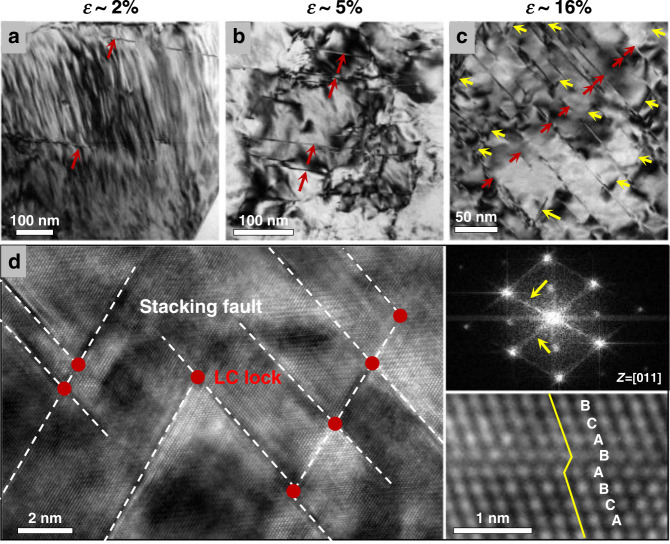
Plastic deformation micro-mechanisms of the CNL alloy. **a** Deformation microstructure at the 2% strain revealing the activation of parallel SFs (red arrow). **b** Activations of more planar SFs (red arrow) at the 5% strain. **c** High-density hierarchical SFs on two {111} slip planes (red and yellow arrows) at the 16% strain. **d** High-resolution TEM image showing the hierarchical SF network (white dash line) and Lomer-Cottrell (LC) locks (red dot) in different intersecting {111} slip systems. The FFT image with crossing diffraction fringes (yellow arrow) reveals the presence of intersected SFs, and an enlarged view of a representative SF is marked by the BCABABCA-stacking sequence.

To understand the underlying mechanisms for the unusual work hardening and large tensile ductility of the ultrahigh-strength CNL alloy, we investigated the dynamic evolution of deformation microstructures at different strains of plastic deformation (Fig. 3). At the strain of ~2% (Fig. 3a), the dislocation slipping is activated in the appearance of extended stacking faults (SFs, red arrow) on the primary {111} planes, which cuts across the FCC/L1_2_ nano-lamellae, indicating an early stage of planar dislocation slip across the coherent lamellar boundaries. With further increasing strain to ~5% (Fig. 3b), the SFs (red arrow) are activated in more grains and with a much higher density as compared with those in the 2% strain state. Further increasing the strain to 16% results in the planar SF-dominated deformation mode with an extremely high-density of SFs on two {111} slip systems (red and yellow arrows) and extensive SF intersections (Fig. 3c). The enlarged view (Fig. 3d) shows that numerous intersected SFs activated on two {111} slip systems, leading to the formation of hierarchical nano-spaced SF networks.

On the basis of these observations, we now discuss the structural origins responsible for the unusual deformation behavior and large ductility of the CNL alloy. First, the presence of extensive SFs suggests that the CNL matrix might have an intrinsically low SF energy. To understand the generation of SFs during plastic deformation, we calculated the SF energy of the CNL matrix using ab initio calculations (Supplementary Fig. 9), where the supercell composition (17Fe-14Co-7Ni-9Cr-1Al) was based on the matrix composition determined by APT. The average SF energy of the FCC lamellar matrix is ~25 mJ m^−2^. This value is in the range of 20–45 mJ m^−2^, within which partial dislocations separation with SF bonds are thermodynamically preferred^26^, thereby making the activation of SFs easy under plastic deformation. For FCC-type materials with low SF energy, a perfect a/2 < 110> unit dislocation tends to dissociate into a pair of a/6 < 112> Shockley partials bonded with the SF during plastic deformation, in contrast to materials with high SF energies in which the dislocation cross-slip and micro-banding are the preferred deformation mode^27^. Therefore, the unusual SF-dominated deformation in the nanolamellar alloys should originate from the low SF energy of the CNL matrix (25 mJ m^−2^), which results in a high propensity of dislocation dissociation into wide SFs in our material, thereby hindering the cross-slip process. Second, the coherent boundaries in the CNL alloy are characterized by continuous crystal lattices, and the orientation of the ordered L1_2_ phase is very close to that of the disordered FCC phase. These structural features facilitate continuous dislocation transmissions across the lamellar boundaries, with no need to change the slip direction^25^. When an applied stress is higher than the yield strength, dislocations can easily transmit across the coherent boundaries, rather than being localized at the boundaries, thereby suppressing the stress concentration due to dislocation accumulations. In addition, the dislocation transmission across the boundaries facilitates long-range dislocation gliding through different L1_2_ and FCC lamellae, which results in the microscopic homogeneous plastic deformation, thereby enhancing the plastic deformation stability and averting an early stage of crack initiation of the CNL alloy. Third, the interaction of the two leading partial dislocations results in the formation of a sessile stair-rod dislocations on a non-slipping {100} plane (Fig. 3d), known as the immobile Lomer-Cottrell (LC) locks. The formation of those high-density LC locks not only act as strong obstacles to effectively pin the dislocation motion, but can also be served as Frank-Read sources for dislocation multiplication^27^, leading to a steady and progressive work hardening of the CNL alloy. Because the formation of SF networks and LC locks is a dynamic process, their density may not be very high at the early stage of the plastic deformation, which may result in a low work-hardening capacity at the early stage of plastic strain. As the deformation proceeds, the density of SF networks and LC locks increases. The LC locks derive their effectiveness in strain hardening from their capability to accumulate dislocations from the two aspects: on the one hand, LC locks serve to stabilize the SF network by pinning lock-forming dislocation segments, because when two dislocations meet to produce an LC lock, four dislocation segments are pinned^28^, and on the other hand, the sessile nature of the stair-rod dislocation in the LC locks enables them to exhibit high structural stability resistant to dissociations and to block other dislocation motion as a barrier, which consequently presents a strong hindering effect on dislocation motion^29^. In addition, the hierarchical SF networks dynamically subdivide the ultrafine grains into even finer sub-grains during deformation (with sizes of ~20 nm at the 16% strain) (Fig. 3d), which can pin the motion of dislocations by decreasing their mean free path (i.e., the dynamic Hall–Petch effect)^30^, thereby further enhancing the work hardening of the CNL alloy. Fourth, APT reveals that the L1_2_ phase in the CNL alloy has a multicomponent composition, which can be regarded as (Ni,Co,Cr,Fe)_3_(Al,Ti). Previous research indicates that the incorporation of Co and Fe in the L1_2_ phase improves its intrinsic ductility^31,32^, whereas the partial substitution of Al by Ti alleviates the environment embrittlement^33^. Furthermore, it has been demonstrated that the multicomponent L1_2_ phase is much stronger and more ductile than binary Ni_3_Al^34^. Therefore, the multicomponent L1_2_ lamellae effectively enhance the strength of the CNL alloys while maintaining high ductilities. Consequently, the combination of the coherent nanolamellar boundaries, high-density immobile LC locks, hierarchical SF networks, and ductile nature of the L1_2_ lamellae substantially enhances the plastic deformation stability and work-hardening capability of the CNL alloy, leading to the large uniform tensile ductility at an ultrahigh-strength level. Upon fracture, the high-density SF interactions and LC locks may act as preferred nucleation sites for microvoids, leading to a homogeneous microvoid coalescence with fine dimples and ductile fracture (cf. Supplementary Fig. 3).

**Fig. 4:**
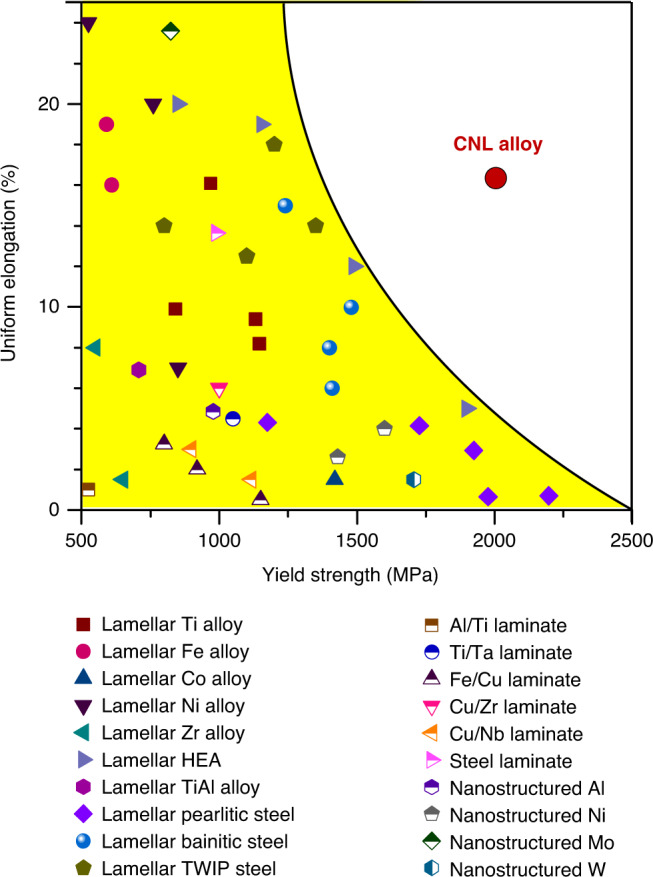
Tensile properties of our CNL alloy compared with those of other materials. The reference materials include lamellar-structured alloys (Ti alloy^35,36^, Fe alloy^37^, Co alloy^38^, Ni alloy^39^, Zr alloy^40^, HEA^41,42^, TiAl alloy^43^, pearlitic steel^44,45^, bainitic steel^46^, and twinning-induced plasticity (TWIP) steel^47^), laminated metal composites (Al/Ti^48^, Ti/Ta^49^, Fe/Cu^50^, Cu/Zr^51^, Cu/Nb^52^, and steel/steel^53^), and nanostructured metals (Al^54^, Ni^55^, Mo^56^, and W^57^). Our CNL alloy (red dot) clearly outperforms other high-performance materials and overcomes the long-standing dilemma of the strength-ductility trade-off.

To illustrate the unusual properties of our CNL alloy, we compare its tensile properties with those of other lamellar materials and high-performance nanostructured materials^35–57^ (Fig. 4). We see that our CNL alloy exhibits an order of magnitude larger uniform elongation than lamellar pearlitic steels while maintaining a tantamount strength^44,45^. Meanwhile, our CNL alloy shows a twofold strength and uniform elongation advantage over lamellar titanium alloys^35,36^. Even when compared with the recently reported lamellar high-entropy alloys (HEAs)^41,42^, our CNL alloy displays a simultaneous 50% increase in both yield strength and uniform ductility. Therefore, our CNL alloy exhibits an unprecedented combination of ultrahigh yield strength and large uniform ductility, which outperforms from other lamellar materials and high-performance nanostructured materials, thereby overcoming the long-standing dilemma of the strength-ductility trade-off. Several major contributions from the unique lamellar architectures account for the superior mechanical properties of the CNL alloy. First, the unique processing approach for tailoring architectures with nanometer-thick lamellae and strong order/disorder lamellar boundaries effectively elevates the yield strength over 2 GPa, reaching to the ultrahigh strength level. Second, the coherent lamellar boundaries eliminate the stress concentration that normally occur in incoherent lamellar materials, thereby enhancing the plastic deformation stability and avoiding an early stage of crack initiation of the CNL alloy. Third, the intrinsically low SF energy (25 mJ m^−2^) of the FCC lamellae promotes the dynamic formation of hierarchical SF networks and high densities of LC locks during plastic deformation, which substantially increases the work-hardening capacity, leading to the superior tensile ductility even at the ultrahigh strength level. Fourth, the fully recrystallized and uniform microstructure promotes the homogeneous deformation, leading to stable mechanical properties of the CNL alloy. This contrasts with non-uniform structured materials, which usually induce inhomogeneous deformation, resulting in large variations of mechanical properties^58^. In addition, the delicate composition design endows our materials with extra advantages compared with other existing ultrahigh-strength materials. For instance, the Cr and Al additions provide a good oxidation resistance of the materials, making them potentially attractive for applications in harsh environments such as in aerospace and aeronautic applications. Furthermore, unlike most conventional nanolamellar materials that are fabricated in the form of thin films/sheets in the micron scale, such as magnetron sputtered and electrodeposited films^59,60^, our CNL materials produced by casting and thermomechanical treatments can potentially be produced in relatively large dimensions (in the mm scale), thus offering a great potential for structural applications in industries.

In summary, we presented an innovative design concept for high-performance materials by engineering nanolamellar architectures, which leads to the development of new bulk nanostructured materials with the unprecedented combination of over 2 GPa yield strength and 16% uniform tensile ductility at ambient temperature. The extraordinary mechanical properties of the CNL materials offer tremendous potential for structural applications in aerospace, automotive, and energy industries. In addition, the fundamental concept of lamellar architecture engineering can be applied to many other metallic materials, including new-generation superalloys, titanium alloys, advanced steels, and HEAs, to achieve enhanced properties for specific applications.

## Methods

### Material preparation

Alloy with nominal composition of Ni_32.8_Fe_21.9_Co_21.9_Cr_10.9_Al_7.5_Ti_5.0_ (at.%), which can also be written as (Ni_1.5_FeCoCr_0.5_)_87.5_Al_7.5_Ti_5.0_, was prepared by arc-melting a mixture of the constituent elements with purity higher than 99.9 wt.% under a Ti-gettered argon atmosphere. Repeated melting was carried out at least four times to ensure the chemical homogeneity. The melted alloys were then drop-cast into copper molds with dimensions of 6 × 15 × 50 mm and 10 × 15 × 50 mm and then homogenized for 2 h at 1150 °C, followed by water quenching. The CNL architecture was produced through controlled thermomechanical treatments consisting of a cold-rolling process for a reduction of 90% and a subsequent tempering treatment for 8 h at 600 °C (referred to as the “CNL alloy”). For comparison, two samples with the same composition as the CNL alloy but different treatment histories were prepared; one was fabricated by cold-rolling the homogenized ingot to a reduction of 90% (referred to as the “severely deformed alloy”), and the other was fabricated by recrystallizing the cold-rolled sample for 3 min at 1150 °C and subsequent tempering for 8 h at 600 °C (referred to as the “conventionally processed alloy”).

**Microstructural characterization**. TEM and STEM observations were conducted on a JEM-2100F microscope operated at 200 kV. The TEM is equipped with EDS for elemental analysis and SAED for structural analysis. TEM specimens were mechanically thinned to ~40 μm, punched to Ф3 mm circle sheets, and then thinned by twin-jet electro-polishing in a solution consisting of 5 vol.% perchloric acid and 95 vol.% alcohol at a temperature of −30 °C with a potential of 24 V. To quantify the observed lamellar thicknesses, TEM images were analyzed by an Image-J software. At least 800 lamellae were measured to obtain a reliable determination. EBSD measurements were performed to characterize the grain structure and orientation distribution using a FEI-SEM with an Oxford detector operated at 30 kV and 0.8 nA. The specimens were prepared by mechanical polishing down to 0.05 μm, followed by electro-polishing in a solution of HNO_3_ (25%) and C_2_H_5_OH (75%) with a voltage of 20 V at −40 °C. Crystal structures were examined by a Rigaku XRD instrument with Cu-K_α_ irradiation. XRD samples were polished using standard mechanical polishing procedures. The *θ*-2*θ* scanning was conducted in the range of 20–100° with a scanning speed of 4°/min. A slow scan in the range of 88–92° with a speed of 0.05°/min was performed to calculate the lattice mismatch (*δ*) at the (311) diffraction peak. The equation can be described as: *δ* = 2(*α*_*L12*_–*α*_*FCC*_)/(*α*_*L12*_ + *α*_*FCC*_), where α refers to the lattice constant for a specific phase. APT characterizations were performed in a CAMECA Instruments LEAP 5000XR local electrode atom probe. The specimens were analyzed in a voltage mode, at a specimen temperature of 70 K, a pulse repetition rate of 200 kHz, a pulse fraction of 0.2, and an ion collection rate of 0.5% ions per field evaporation pulse. Needle-shaped specimens required for APT were fabricated by lift-outs and annular milled in an FEI Scios focused ion beam/scanning electron microscope (FIB/SEM). Imago Visualization and Analysis Software version 3.8 was used for three-dimensional reconstructions and data analysis. The obtained compositions were used to evaluate the volume fraction of the FCC and L1_2_ lamellae by using the level rule. The equation can be described as: *ƒ*^*L12*^ = (*c*^*nominal*^_*i*_–*c*^*FCC*^_*i*_)/(*c*^*L12*^_*i*_–*c*^*FCC*^_*i*_), where *c*^*nominal*^_*i*_, *c*^*L12*^_*i*_, and *c*^*FCC*^_*i*_ refers to the atomic ratio of element *i* in the nominal alloy composition, L1_2_, and FCC phases, respectively. Plotting the concentration for each element species, and fitting the data to a linear relationship yields a slope, which is equal to the volume fraction of the L1_2_ phase.

**Mechanical tests**. Room temperature tensile properties were evaluated using an MTS mechanical testing system with a strain rate of 10^−3^ s^−1^. Dog-bone shaped tensile specimens with a gauge length of 12.5 mm and a cross-section area of 3.2 × 0.7 mm^2^ were prepared using electrical-discharge machining. During tensile testing, a high-resolution strain extensometer was attached to the gauge length section, which ensures an accurate measurement of the yielding behavior and tensile ductility. At least three tensile samples for each condition were tested to obtain statistically valid results. In addition, larger-sized tensile samples with a gauge length of 25 mm and a gauge width of 5 mm were tested to verify the initial results, and the resulted mechanical properties are displayed in Supplementary Fig. 10. Fracture surfaces of tensile samples were examined by the FEI-SEM.

### Thermodynamic and ab initio calculations

Thermodynamic calculations were performed by the Thermo-Calc 3.0.1 software with a Ni-based database (TTNi8). Ab initio calculation was performed using the Vienna ab initio simulation package (VASP)^61,62^ with the projector augmented wave^63,64^ method and the Perdew–Becke–Erzenhof^65^ exchange-correlation functional. A plane wave cutoff energy of 400 eV and a k-point mesh of 9 × 3 × 1 were used, and magnetism was considered. A vacuum layer of 25 Å was set in the 48 atoms FCC supercell, which was built by special quasirandom structure method^66^. The supercell composition was 17Fe-14Co-7Ni-9Cr-1Al, which is based on the matrix composition determined by APT. Supplementary Fig. 9 shows the method to build a SF in the FCC supercell, which has 12 different atomic layers in the direction perpendicular to the SF plane. Because the arrangements of atoms are disordered in the FCC phase, each atomic layer in the supercell was moved to build 12 derivative supercells containing different SF planes. The mean value of the 12 calculated results was taken as the average SF energy of the FCC phase.

**Theoretical calculations of strengthening responses**. It was documented that a Hall–Petch relationship also describes the yield strength of dual-phase nanolamellar materials^25^. In these materials, dislocations initially start to propagate inside the soft phase and pile-up at interphase boundaries. The yield occurs when the leading dislocation in a pile-up can overcome the barrier strength and transmit slip across the boundary. Thus, the parameters controlling the strength are the thickness and physical properties of the soft lamellae^25^. In the FCC/L1_2_ CNL architecture, initial plastic deformation occurs in the soft FCC lamellae; thus, the parameters controlling the yield strength is the thickness and physical properties of the FCC lamellae. The strengthening contributed by the FCC/L1_2_ boundaries can be described by $\Delta \sigma _l = k_l(\lambda )^{ - 0.5}$, where *k*_*l*_ is the Hall–Petch coefficient and *λ* is the average thickness of the FCC lamellae. The Hall–Petch coefficient *k*_*l*_ can be calculated by^67^:

1 $$k_l = \left( {\frac{{n^2G^2b^2}}{{8\lambda }}} \right)^{1/2}$$

where *n* = ~4.5 is the number of dislocations crossing the same lamellae (obtained through TEM analysis of the 2% and 5% strained samples), *G* = 84 GPa is the shear modulus of the FCC phase^68^, $b = \sqrt 2 a/2$ = 0.255 nm is the magnitude of the Burgers vector of the FCC phase, *a* = 0.3585 nm is the lattice constant of the FCC phase obtained from XRD, *d* = ~37 nm is the average lamellar thickness of the FCC lamellae. Using these data, the strengthening contribution by the nanolamellar boundaries was estimated to be ~1.0 GPa, which accounts for approximately a half of the total yield strength, providing the dominant contribution to the macroscopic yield strength.

The ultrafine grain structure also contributes considerably to the yield strength. The grain boundary strengthening can be described using the well-known Hall–Petch relationship $\Delta \sigma _g = k_g(d)^{ - 0.5}$, where *k*_*g*_ = ~516 MPa μm^0.5^ is the Hall–Petch coefficient, measured from an alloy having the composition of the FCC lamellae in the CNL alloy (Supplementary Fig. 11), and *d* = ~390 nm is the average grain size of the CNL alloy. The strengthening due to the ultrafine grains was calculated to be 0.8 GPa. Therefore, the total strengthening contributed by the lamellar and grain boundaries is ~1.8 GPa, accounting for as high as 90% of the total yield strength.

## Supplementary information

Supplementary Information

## Data Availability

The data that support the findings of this study are available from the corresponding authors on reasonable request.

## Code availability

The VASP is a proprietary software available for purchase at https://www.vasp.at/.
