# Supplementary material for: Ultrahigh strength and ductility in newly developed materials with coherent nanolamellar architectures
Source: Nat Commun. 2020 Dec 7;11:6240. doi: 10.1038/s41467-020-20109-z (PMC7721903; doi:10.1038/s41467-020-20109-z)
Supplement: Supplementary file 1 — Supplementary Information [file 41467_2020_20109_MOESM1_ESM.pdf]

Supplementary information for

Ultrahigh strength and ductility in newly developed materials with coherent nano-lamellar architectures

Lei Fan[1], Tao Yang[2], Yilu Zhao[2], Junhua Luan[2], Gang Zhou[3], Hao Wang[3], Zengbao Jiao[1,*] & Chain-Tsuan Liu[2,*]

[1] Department of Mechanical Engineering, The Hong Kong Polytechnic University, Hong Kong, China

[2] Department of Materials Science and Engineering, City University of Hong Kong, Hong Kong, China

[3] Shi-changxu Innovation Center for Advanced Materials, Institute of Metal Research, Chinese Academy of Sciences, Shenyang 110016, China

* *Corresponding authors: zb.jiao@polyu.edu.hk; chainliu@cityu.edu.hk*

This file includes:

Supplementary Figures 1-11

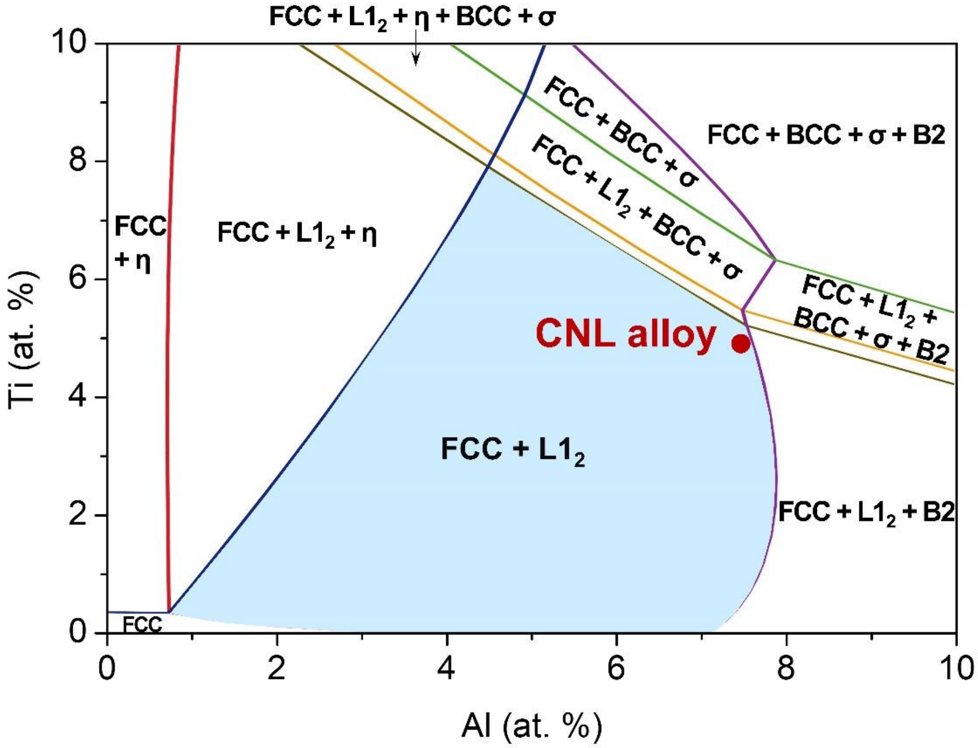

Supplementary Fig. 1 Thermodynamic calculations of the isothermal section of $(Ni_{1.5}CoFeCr_{0.5})_{100\text{-}x\text{-}y}Al_xTi_y$ (at.%) at 600 ˚C. The Ni-Co-Fe-Cr-Al-Ti system has a large dual-phase region consisting of disordered-FCC and ordered-$L1_2$ phases with a small lattice mismatch, satisfying the requirement for the formation of coherent boundaries. The red spot indicates the position of the CNL alloy ($Ni_{32.8}Fe_{21.9}Co_{21.9}Cr_{10.9}Al_{7.5}Ti_{5.0}$).

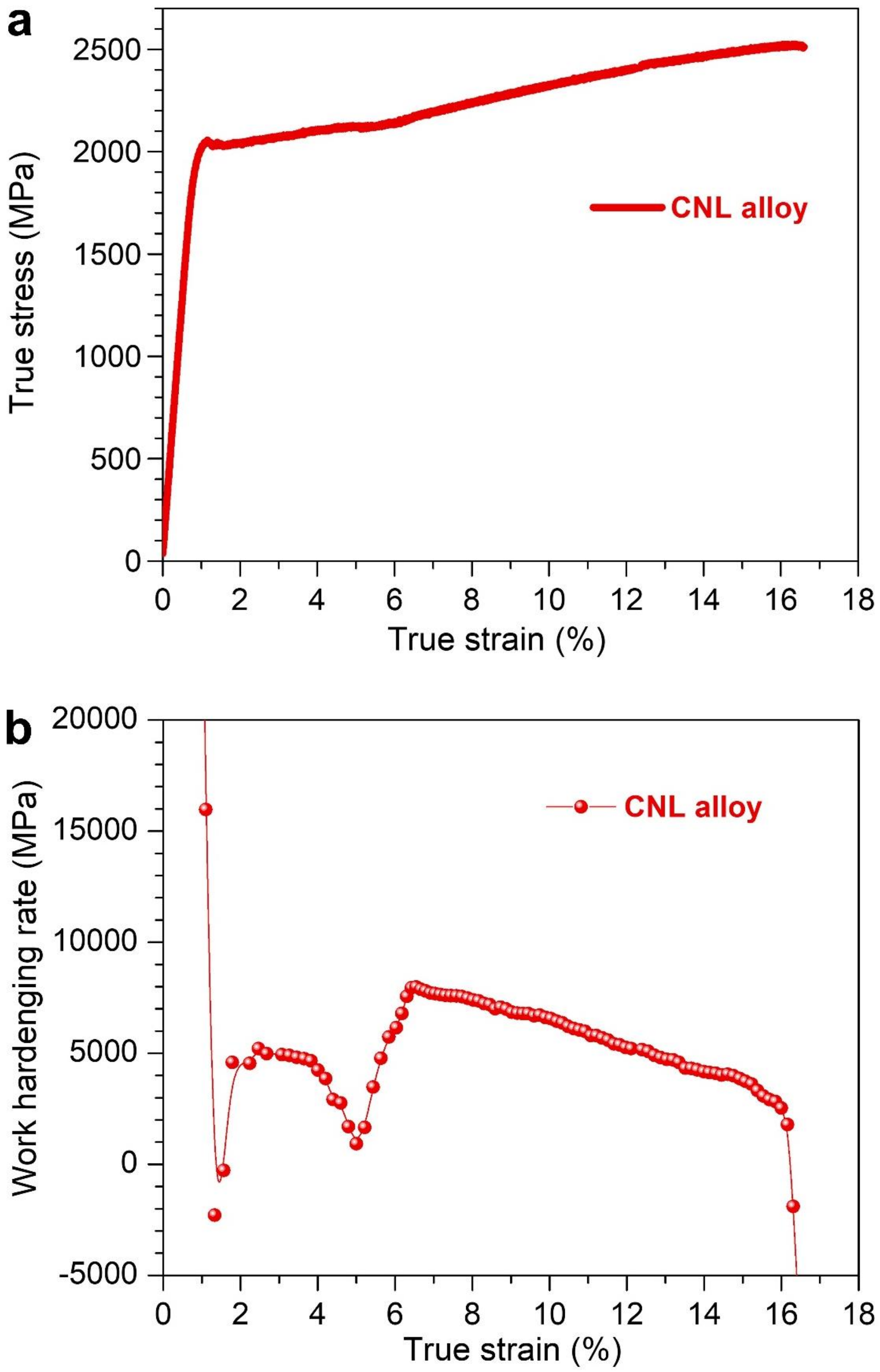

Supplementary Fig. 2 Tensile behavior of the CNL alloy. a True stress-strain curve. **b** Work hardening rate as a function of true strain.

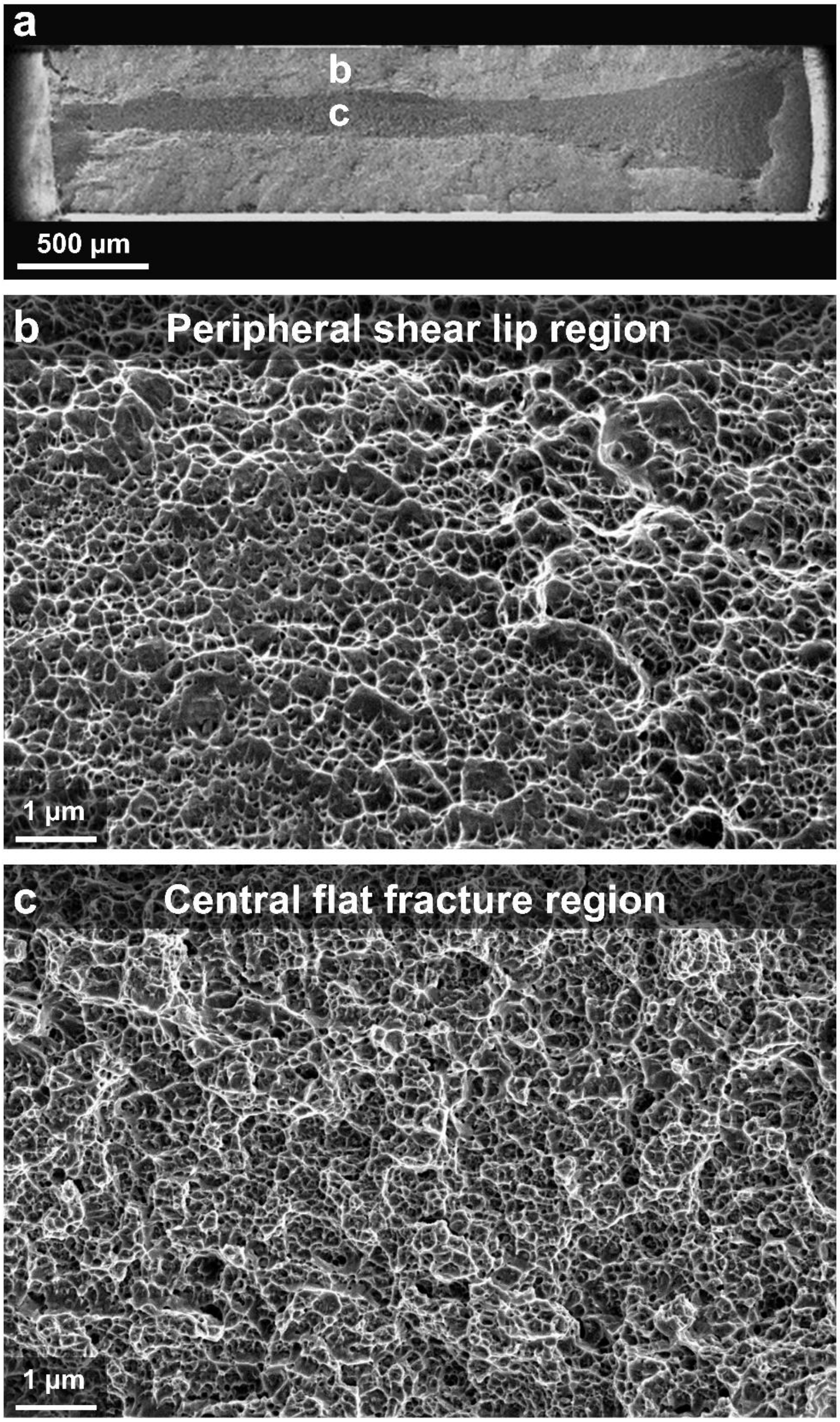

Supplementary Fig. 3 Fracture surfaces of the CNL alloy. a Overview of the fracture surface. **b** High magnification of the peripheral shear lip region. **c** High magnification of the central flat fracture region. Both regions show a plenty of fine dimples, indicating a characteristic mode of a ductile fracture.

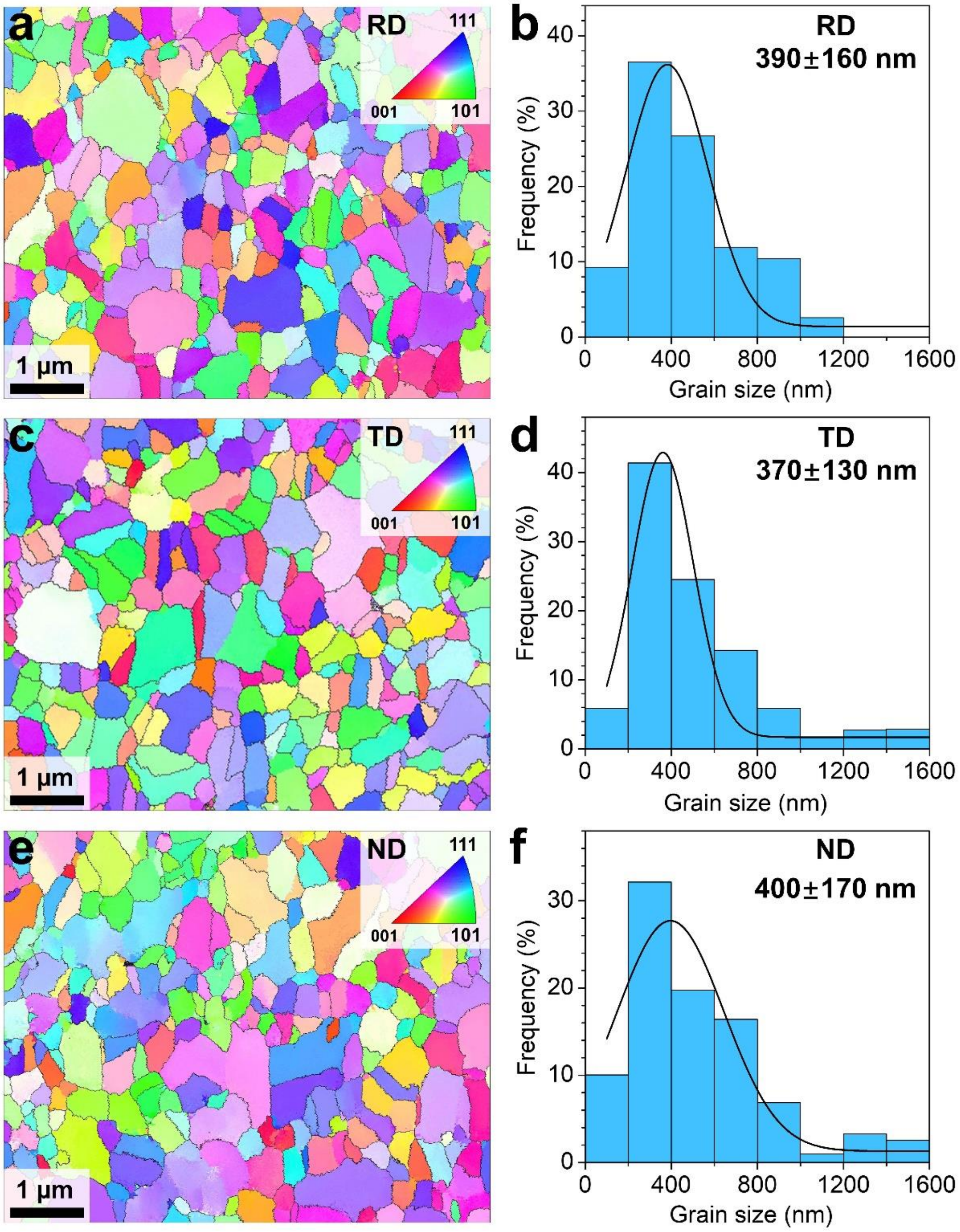

Supplementary Fig. 4 EBSD images and grain size distributions of the CNL alloy along the different directions. a, b Rolling direction (RD). **c, d** Transverse direction (TD)**. e, f** Normal direction (ND). The CNL alloy exhibits a uniform distribution of equiaxed ultrafine grains with an average size of ~390 nm and random orientations.

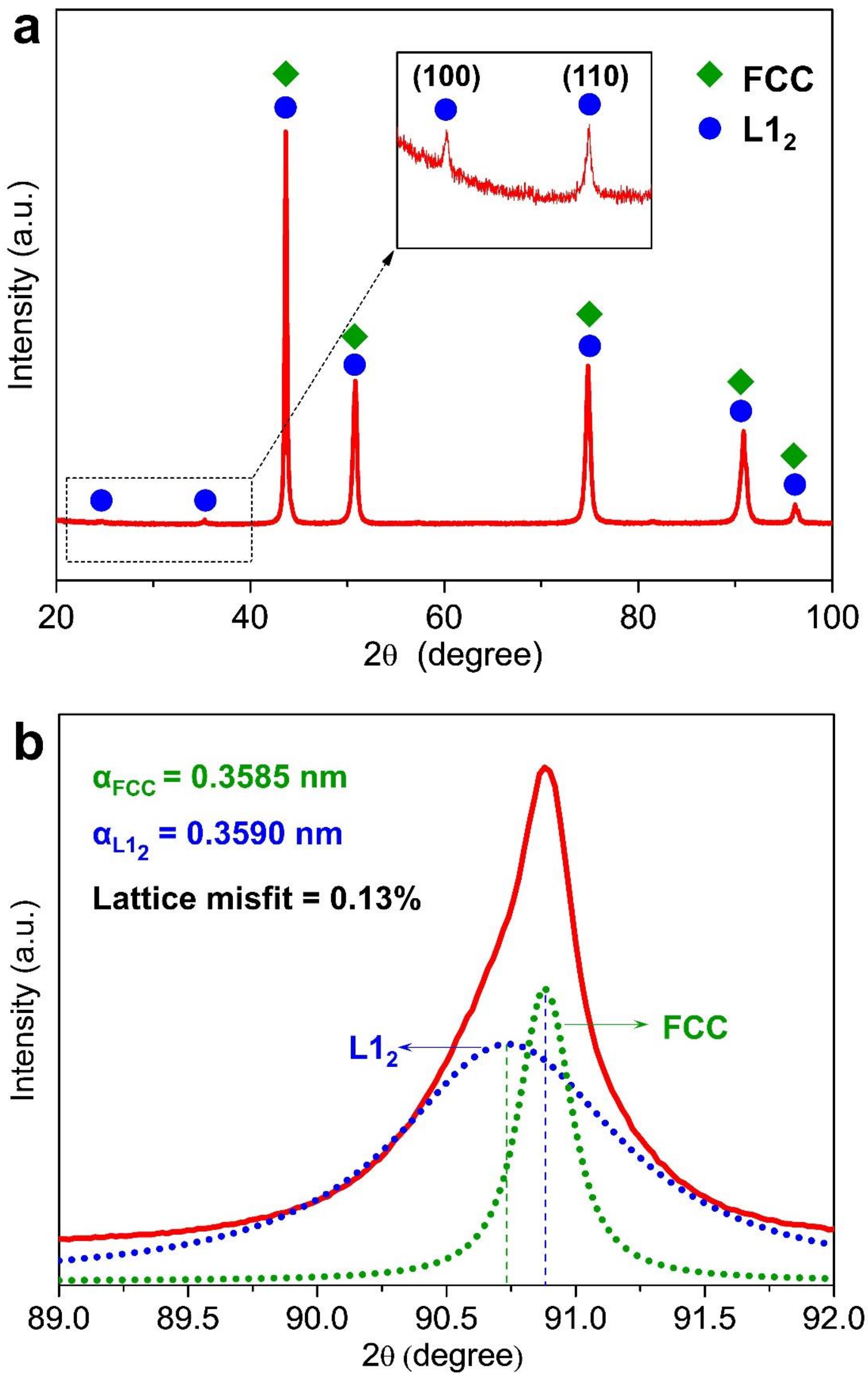

Supplementary Fig. 5 XRD patterns of the CNL alloy. a The diffraction pattern showing the co-existence of FCC and $L1_2$ phases. **b** Deconvolution of the (311) diffraction peak for determining the lattice parameter of the FCC and $L1_2$ phases.

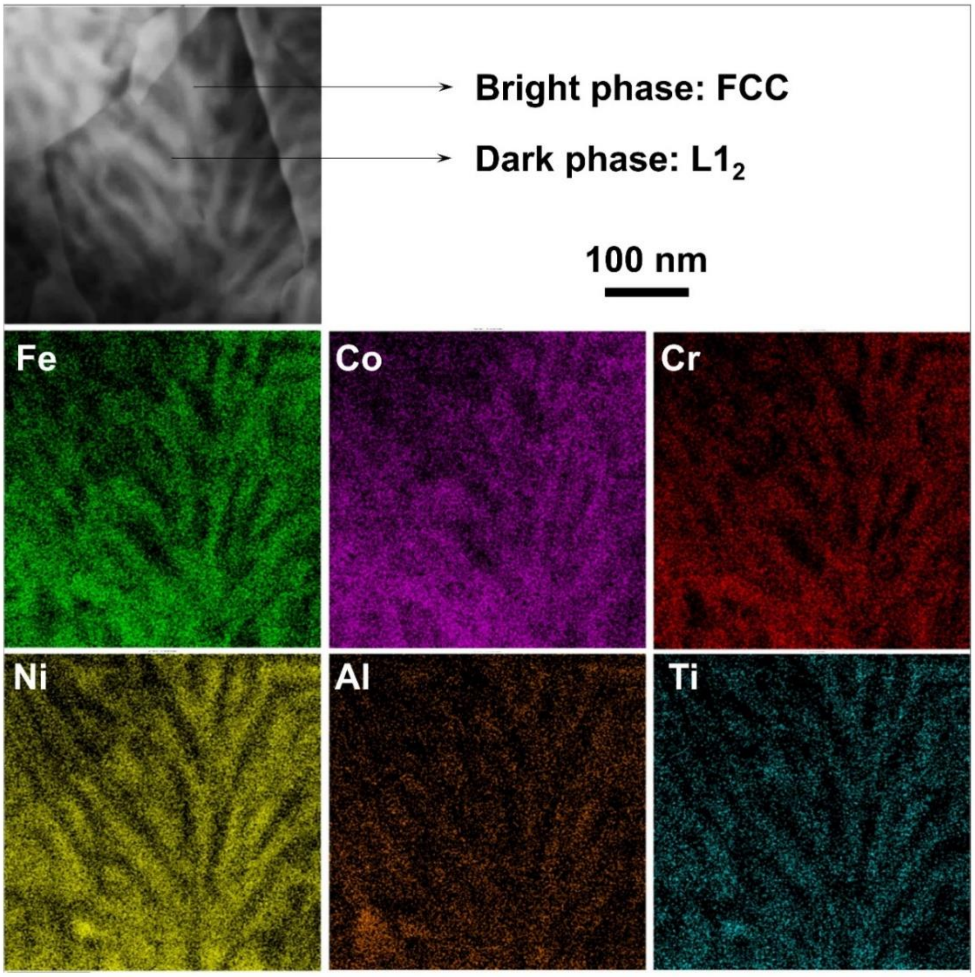

Supplementary Fig. 6 STEM-EDS mappings of the CNL alloy. The elemental maps illustrate that Fe, Co, and Cr partition to the FCC phase and Ni, Al, and Ti partition to the $L1_2$ phase, which is inconsistent with the APT characterization.

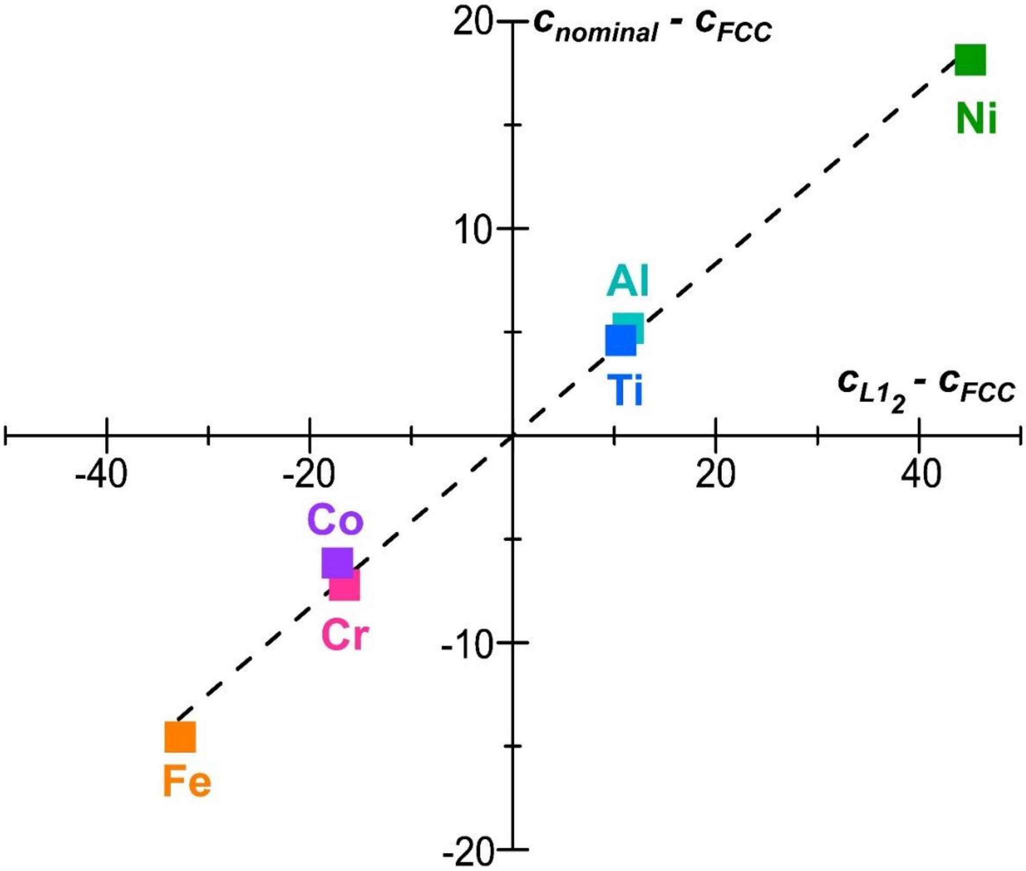

Supplementary Fig. 7 Determination of phase volume fractions of the CNL alloy by using the lever rule. The APT concentrations of Fe, Co, Cr, Ni, Al, and Ti were used in the analysis. The volume fraction of the FCC and $L1_2$ phases were estimated to be approximately 56% and 44%, respectively.

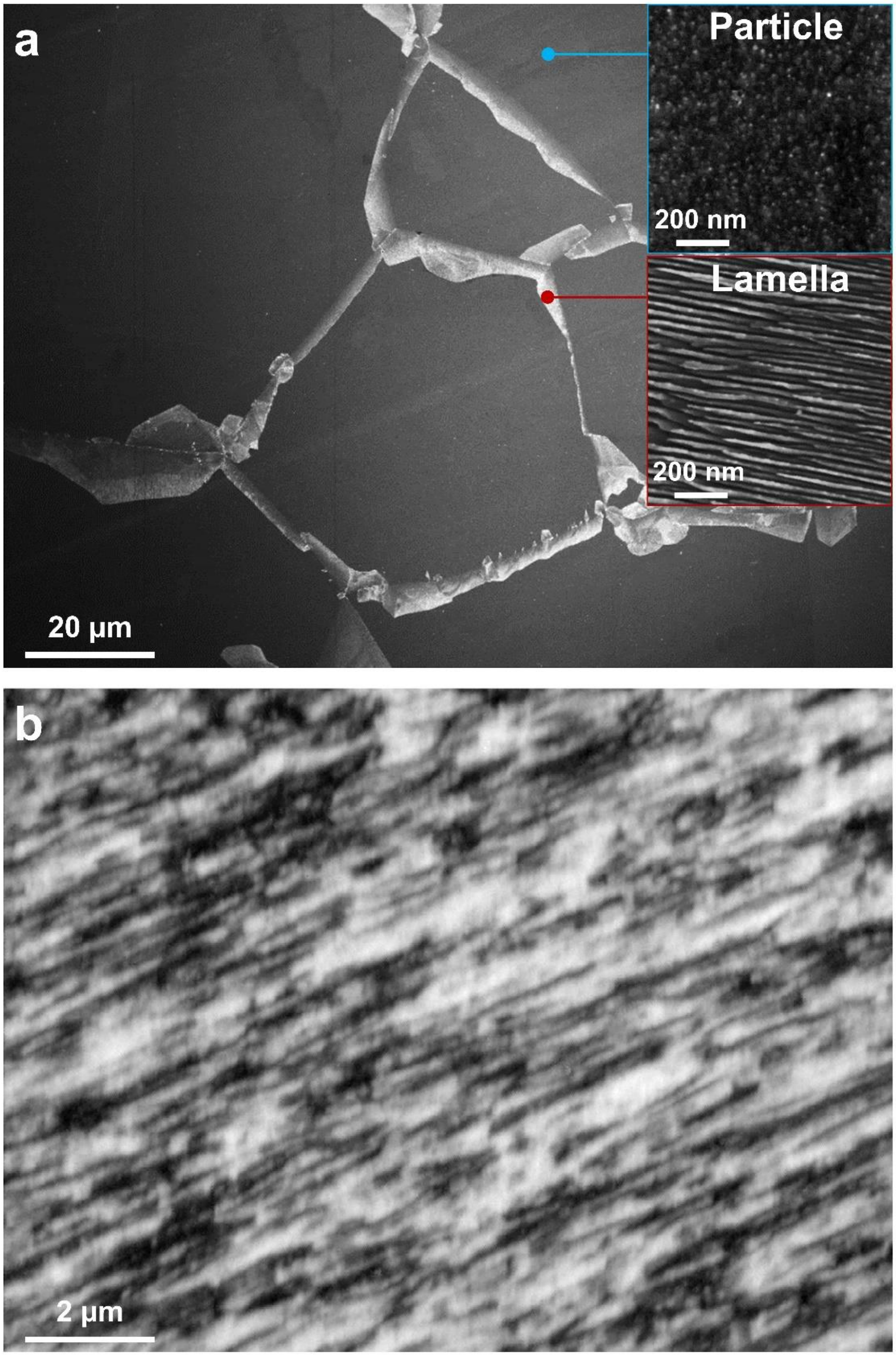

Supplementary Fig. 8 Microstructures of the reference alloys. a Conventionally processed alloy. **b S**everely deformed alloy.

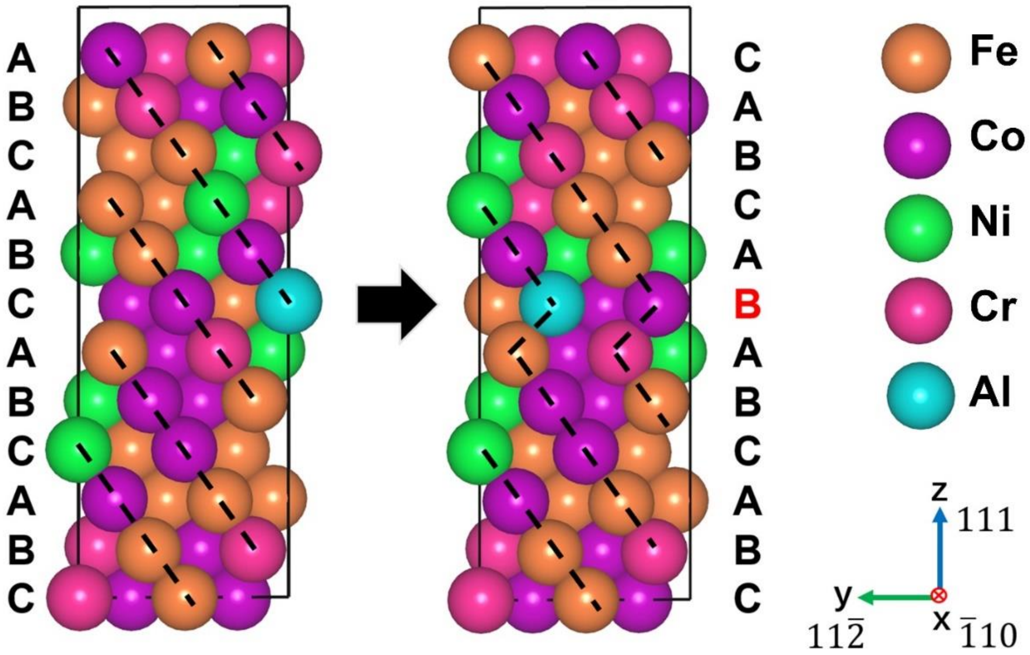

Supplementary Fig. 9 Atomic configurations of the original FCC structure and defective structure with intrinsic stacking faults. The FCC supercell has 12 different atomic layers in the direction perpendicular to the stacking fault plane, and 12 derivative supercells containing different SF planes were built by moving each layer. Descriptions for the determination of SF energy by ab initio calculations are summarized in Methods.

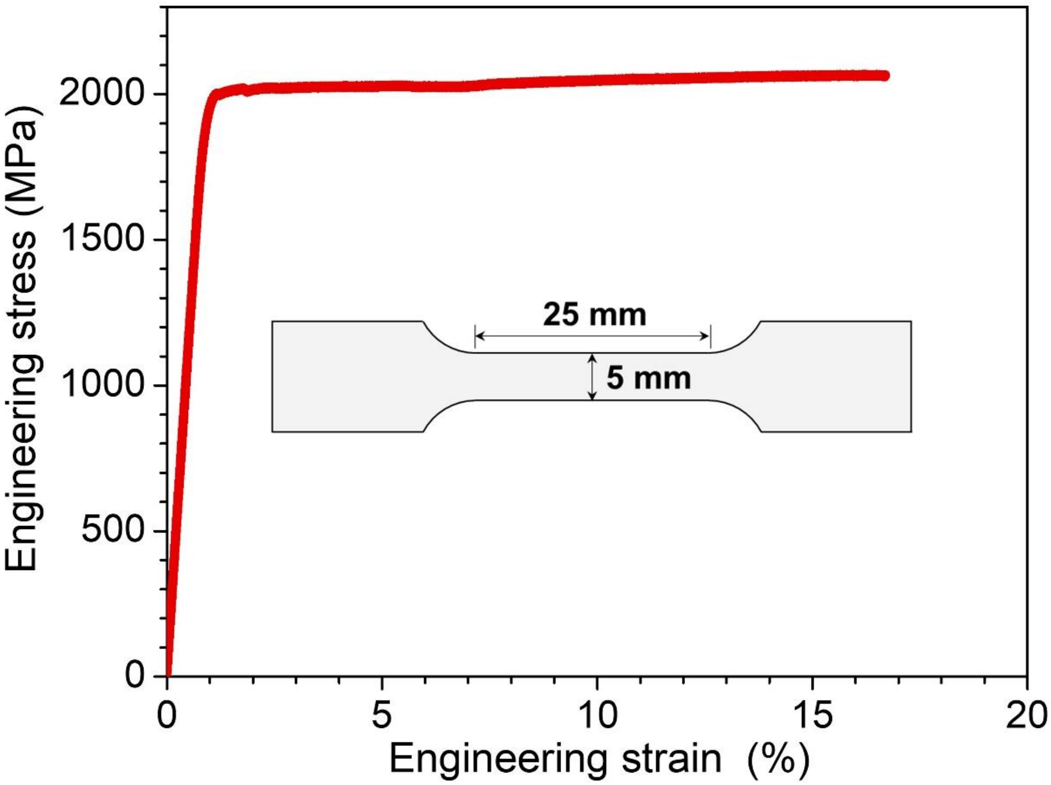

Supplementary Fig. 10 Tensile engineering stress-strain curve of the CNL sample with a gauge length of 25 mm and a gauge width of 5 mm. The mechanical properties of the large-sized samples are highly comparable to our initial results.

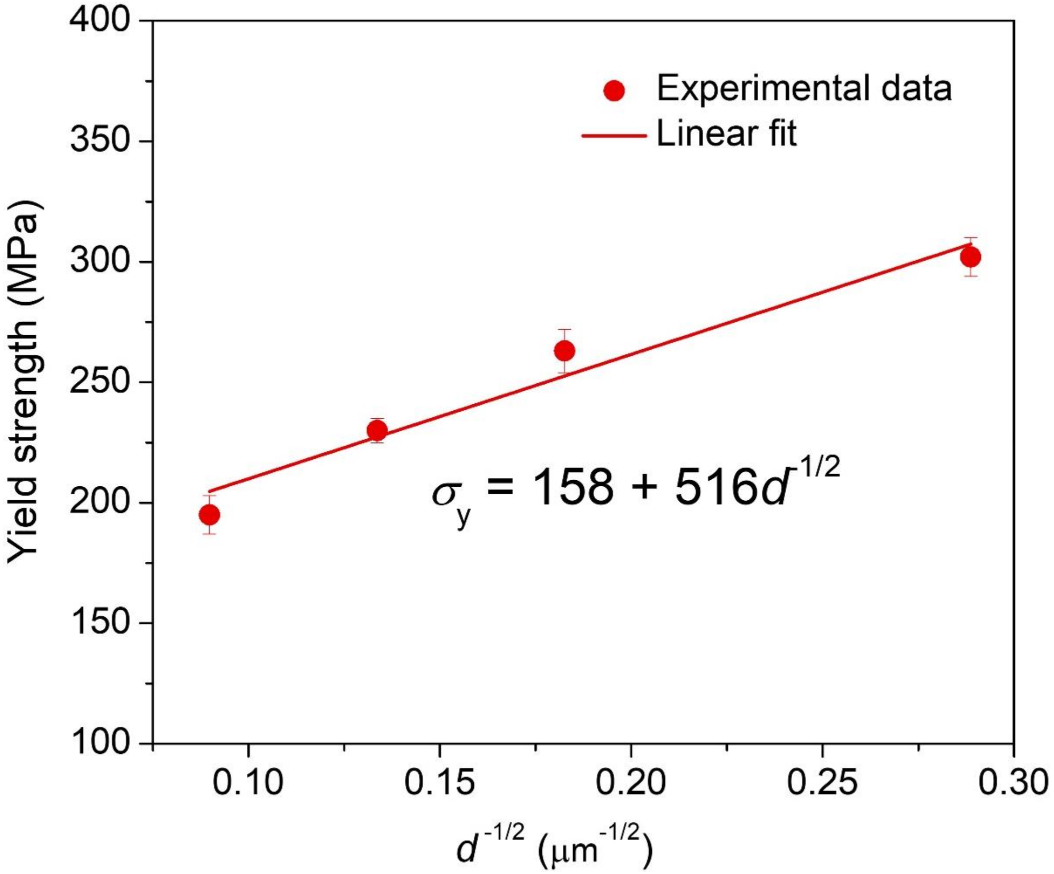

Supplementary Fig. 11 Plot of the yield strength as a function of the inverse square root of mean gain size of the alloy having the composition of the FCC matrix in the CNL alloy.
